# Multi-fluid, multi-omics signatures of insulin resistance and incident type 2 diabetes among Puerto Rican adults

**DOI:** 10.3389/fendo.2025.1699656

**Published:** 2025-12-04

**Authors:** Tong Xia, Zicheng Wang, Teja Lakamraju, Danielle E. Haslam, Saravanan Thangarajan, David T. W. Wong, Liming Liang, Kaumudi Joshipura, Meir J. Stampfer, Frank B. Hu, Kyu Ha Lee, Shilpa N. Bhupathiraju

**Affiliations:** 1 Channing Division of Network Medicine, Department of Medicine, Brigham and Women’s Hospital and Harvard Medical School, Boston, MA, United States; 2 Department of Nutrition, Harvard T. H. Chan School of Public Health, Boston, MA, United States; 3 Department of Epidemiology, Harvard T. H. Chan School of Public Health, Boston, MA, United States; 4 Department of Biomedical Engineering, University of Connecticut, Storrs, CT, United States; 5 Department of Global Health and Social Medicine, Harvard Medical School, Boston, MA, United States; 6 School of Dentistry, University of California, Los Angeles, Los Angeles, CA, United States; 7 Department of Biostatistics, Harvard T. H. Chan School of Public Health, Boston, MA, United States; 8 Bagchi School of Public Health, Ahmedabad University, Ahmedabad, Gujarat, India

**Keywords:** plasma, saliva, proteomics, metabolomics, diabetes

## Abstract

**Introduction:**

Previous studies have examined the prediction of insulin resistance and type 2 diabetes (T2D) using plasma or saliva omics, but none have combined metabolomics and proteomics from multiple biofluids, such as plasma and saliva. Among Puerto Rican adults, a high-risk population with health disparities, we sought to determine whether adding saliva improves T2D prediction over plasma alone.

**Methods:**

In this pilot matched case–control study within the San Juan Overweight and Obese Adults Longitudinal Study (SOALS), we analyzed baseline samples from 40 healthy participants, 20 of whom developed T2D at follow-up (year 3) and 20 age- and sex-matched controls. We profiled 7,595 proteins in plasma and saliva (SomaScan) and 1,051 plasma and 635 saliva metabolites [ultra-high-performance liquid chromatography–tandem mass spectrometry (UHPLC–MS/MS) and gas chromatography–mass spectrometry (GC–MS); Metabolon, Inc.] for analysis. We evaluated nine omics signatures combining biofluid (plasma, saliva, or both) and omics (metabolomics, proteomics, or both). Nested elastic net regression with leave-one-out cross-validation identified insulin resistance signatures, and receiver operating characteristic (ROC) curves [area under the curve (AUC)] assessed their predictive performance for T2D. We used multivariable conditional logistic regression to evaluate associations between omics scores and incident T2D.

**Results:**

The strongest T2D prediction was observed for plasma proteomics and multi-omics, multi-fluid proteomics, and multi-omics signatures (AUCs: 0.80–0.83). Saliva proteomics, metabolomics, and multi-omics, along with plasma metabolomics and multi-fluid metabolomics, exhibited limited prediction (AUCs: 0.51–0.67). Plasma proteomics, multi-omics, and multi-fluid multi-omics were positively associated with T2D [hazard ratios (HRs): 3.00–3.68].

**Conclusion:**

Plasma proteomic signatures provided the strongest T2D prediction. Adding saliva data did not improve predictive performance of plasma data.

## Introduction

Type 2 diabetes (T2D) is a major public health concern (1). Racial and ethnic disparities in T2D prevalence are well documented in the US (1, 2). Hispanics, the largest minority group in the US, bear a disproportionate burden of T2D, with higher prevalence of both diagnosed (13.0% *vs*. 8.9%) and undiagnosed (4.6% *vs*. 2.3%) T2D compared to non-Hispanic white persons (3). Among Hispanic subgroups, Puerto Ricans have the highest T2D prevalence at 29.1% (4). These disparities underscore the importance of early screening strategies to prevent disease onset or delay T2D progression.

Metabolomics has been widely used to uncover metabolic alterations in disease states (5–8). However, it only captures part of the biological complexity. Advances in proteomic technologies over the past decade have enabled high-throughput exploration of the proteome, facilitating the discovery of novel diagnostic biomarkers (9–14). Because proteomics measures the end product of the gene expression cascade, proteins, it provides critical insights into biological function and disease mechanisms. When combined with metabolomics, multi-omics approaches can provide a comprehensive view of the molecular changes underlying T2D.

Although plasma is the most studied biofluid, saliva offers a potential alternative due to its non-invasive, safe, and convenient collection methods. Moreover, human saliva is a rich reservoir of metabolites and proteins. While previous studies have investigated T2D-related metabolomic signatures in plasma (5–7) and saliva (7), as well as proteomic profiles in plasma (9–11) and saliva (12–14), only one study to date has utilized multi-fluid metabolomics for T2D prediction (8). Additionally, no prior studies have integrated metabolomics and proteomics across both plasma and saliva to assess their collective utility in predicting T2D.

In this predictive modeling study, we aimed to (1) characterize proteomic, metabolomic, and multi-omics signatures of insulin resistance in plasma, saliva, and across multi-fluids; and (2) assess their predictive capability and associations with incident T2D in a high-risk adult population with overweight and obesity living in Puerto Rico.

## Materials and methods

### Study population

The San Juan Overweight Adults Longitudinal Study (SOALS) is a longitudinal, population-based cohort of 1,300 Puerto Rican adults (aged 40–65 years) who were overweight or obese [body mass index (BMI) ≥ 30 kg/m^2^] and without a clinical diagnosis of T2D from the San Juan area, which began in 2011. Additional details on the study design have been provided previously (15). Of the 1,300 enrolled participants, 911 had baseline saliva and plasma metabolomics measurements. The current study is a pilot nested case–control study using baseline biospecimens for proteomics and metabolomics. From participants free of T2D at baseline, we randomly selected 20 incident T2D cases identified during 3 years of follow-up and 20 controls matched 1:1 on age and sex. None of the 40 selected participants were taking antihypertensives or lipid-lowering drugs at study baseline. The University of Puerto Rico Institutional Review Board approved the study, and all participants provided written consent before enrolling in the study.

### Assessment of insulin resistance and selection of T2D cases and controls

Fasting blood samples were collected at baseline and follow-up in silicone-coated sterile EDTA blood collection tubes (Becton Dickinson Vacutainer Systems, NJ), centrifuged, and stored at −80 °C. Serum insulin concentrations were measured using commercially available chemiluminescence assays (Tosoh analyzer; Tosoh Bioscience, San Francisco, CA, USA), with intra-assay and inter-assay coefficients of variation of 1.49% and 4.42%, respectively. Insulin resistance was estimated using the Homeostatic Model of Insulin Resistance (HOMA-IR), computed as fasting insulin (μU/L) multiplied by fasting glucose (nmol/L)/22.5. T2D was defined using the American Diabetes Association criteria (16). Participants with T2D included those with a fasting plasma glucose (FPG) concentration ≥7.0 mmol/L (126 mg/dL), or a 2-h plasma glucose concentration ≥11.1 mmol/L (200 mg/dL) during an oral glucose tolerance test (OGTT), or glycated hemoglobin (HbA1c) ≥48 mmol/mol (6.5%), or were currently taking glucose-lowering medication.

### Metabolomics and proteomics profiling

Unstimulated saliva samples were collected under a standardized protocol (17) after at least 2 h abstention from food, drink, tobacco, or oral hygiene. Supernatants (5 mL) were centrifuged, treated with protease inhibitors, and stored at −80 °C. Laboratory personnel were blinded to case–control status. Plasma and saliva metabolomics were analyzed by Metabolon, Inc. using ultra-high-performance liquid chromatography–tandem mass spectrometry (UHPLC–MS/MS) and gas chromatography–mass spectrometry, identifying 1,278 plasma and 769 saliva metabolites. Metabolomics quality control incorporated multiple measures: (i) pooled study matrices prepared from all experimental samples to monitor analytical reproducibility; (ii) extracted water process blanks to detect carryover and background contamination; and (iii) a cocktail of internal/quality-control standards to track instrument performance. Sample injections were randomized to mitigate batch and drift effects, and post-acquisition normalization was applied to correct inter-day variation due to instrument tuning. Proteomic analyses were conducted using the SomaScan Assay v5.0, which identified 7,596 proteins across plasma and saliva samples. SOMAmer^®^ reagents enabled protein quantification via DNA detection technology. The v5.0 assay measures ~11,000 human proteins spanning >10 orders of magnitude in concentration with high sensitivity (<1 pg/mL) and a manufacturer-reported median coefficient of variation (CV) < 5%. To control batch effects and ensure quality, each 96-well plate (≈85 samples/plate) included 5 pooled plasma calibrators, 3 pooled plasma QC replicates, and 3 buffer (no-protein) replicates, plus 12 hybridization controls and 296 non-human SOMAmers to monitor assay and readout performance. Data were processed using SomaLogic’s standard normalization/batch-correction pipeline, hybridization control normalization, plate scaling (calibrators), median normalization, calibration, and Adaptive Normalization by Maximum Likelihood (ANML), to correct sample-, plate-, and SOMAmer-level variability. Technical reproducibility was monitored via the calibrator and QC replicates across plates/runs; consistent with platform specifications, precision supported singlet processing. Cross-platform validation of SOMAmer-based measurements has been demonstrated against orthogonal technologies [e.g., LC–MS/MS, Olink, Luminex, and enzyme-linked immunosorbent assay (ELISA)], supporting the analytical validity of the assay. Among the identified metabolites and proteins, missing values were imputed as half the minimum value, and inverse normal transformations were applied. A total of 1,051 plasma metabolites, 635 saliva metabolites, and 7,595 proteins in both saliva and plasma were available for analysis.

### Ascertainment of covariates

In-person interviews were conducted at baseline and 3-year follow-up. Information on baseline covariates was collected using validated questionnaires. Comprehensive demographic and health-related information included age (continuous), sex (male/female), education (below high school and high school or higher), annual income (less than $20,000 and $20,000 or above), smoking status (never, former, and current), alcohol intake (grams per week), family history of diabetes (yes/no), medication use (yes/no), and mouthwash use (yes/no). Physical activity was quantified in terms of metabolic equivalent (MET) scores derived from a questionnaire that evaluated the frequency and type of physical activity (18). Perceived stress was assessed using the Spanish version of the Perceived Stress Scale (19). BMI was calculated as the weight divided by the square of the height (kg/m^2^). Waist circumference was measured at the level of umbilicus using a non-elastic tape by trained staff.

### Statistical analysis

Baseline characteristics were summarized using means and standard deviations for continuous variables and frequencies with percentages for categorical variables. We used chi-square tests to compare categorical variables and two-sample *t*-tests for continuous variables between cases and controls. When expected cell counts were less than 5, Fisher’s exact test was applied. We applied nested elastic net regression using the *glmnet* R package to derive nine omics profiles of insulin resistance: plasma metabolomics, plasma proteomics, plasma multi-omics (metabolomics and proteomics), saliva metabolomics, saliva proteomics, saliva multi-omics, multi-fluid (saliva and plasma) metabolomics, multi-fluid proteomics, and multi-fluid multi-omics. To minimize overfitting, we used a nested cross-validation framework: in the outer loop, we performed leave-one-out cross-validation (LOOCV), training the model on 39 participants and generating predictions for the left-out individual. In the inner loop, we used 10-fold cross-validation within the training set to select the optimal regularization parameter (λ), specifically choosing the value of λ that minimized cross-validation error (λ_min). This rigorous two-level approach ensured that model selection and evaluation were fully independent, reducing bias and overfitting risk in this small-sample setting. Weights were assigned to each metabolite and protein to reflect their contribution in predicting baseline HOMA-IR concentrations. Omics scores were calculated by multiplying the weights by their respective values. We evaluated the model performance of the nine omics signature scores to predict incident T2D by constructing receiver operating characteristic (ROC) curves and their area under the curve (AUC) values. To further assess robustness, we performed resampling on the fixed LOOCV-derived prediction scores. A permutation test (*B* = 2,000) permuted case–control labels within matched sets to obtain an empirical *p*-value for AUC. A bootstrap (*B* = 2,000) sampled matched sets with replacement to compute 95% CIs for AUC conditional on the fixed scores.

We used conditional logistic regression to evaluate associations between omics-derived signatures and incident T2D in a nested case–control design within the SOALS cohort. Each case was matched to controls by age and sex, and matching was incorporated through risk-set stratification [strata(Obs)], such that all comparisons were made within matched sets. Robust standard errors were computed using a cluster term for participant ID [cluster(ID)]. Model 1 was a crude conditional model that accounted for the matching factors (age and sex) by design but included no additional covariates. Model 2 additionally adjusted for potential confounders using an outcome-free, low-dimensional confounder score. Specifically, we constructed a principal component (PC)-based score derived from the following variables: smoking status, income, education, psychosocial stress, physical activity, alcohol consumption, mouthwash use, family history of diabetes, and BMI. We summarized potential confounders into outcome-free PC scores computed from the covariate matrix (numeric variables standardized; indicator variables unscaled). Hazard ratios (HRs) and 95% confidence intervals (CIs) were estimated from the conditional likelihood, which is equivalent to the Cox partial likelihood under incidence-density sampling. All statistical analyses were conducted using R (version 4.1.0 or higher; https://cran.r-project.org). To account for multiple comparisons, we controlled the false discovery rate (FDR) at 0.05 using the Benjamini–Hochberg (B–H) procedure.

## Results

Participants in both case and control groups were predominantly female (60.0%, 95% CI: 38.7%–78.1% for both groups) with a mean age of 49 years. Compared to controls, cases had higher baseline concentrations of fasting insulin (16.08 *vs*. 8.92 µIU/mL), glucose (96.30 *vs*. 86.00 mg/dL), HbA1c (5.95 *vs*. 5.48%), HOMA-IR (3.90 *vs*. 1.93 mg/dL), and triglyceride (204.75 *vs*. 131.80 mg/dL) (**Table 1**).

**Table 1:** Baseline characteristics of participants in the nested case–control study of SOALS.

Characteristic	Control, *n* = 20	Type 2 diabetes cases, *n* = 20	*P*-value
Female, *n* (%)	12 (60.0)	12 (60.0)	1.00
Age, years	49.25 (5.73)	49.10 (6.19)	0.94
Having family history of diabetes, *n* (%)	7 (35.0)	11 (55.0)	0.34
Education level, *n* (%)			0.35
<High school	2 (10.0)	1 (5.00)	
High school graduate	12 (60.0)	9 (45.0)	
Some college	2 (10.0)	7 (35.0)	
College degree	4 (20.0)	3 (15.0)	
Income <$20,000, *n* (%)	15 (75.0)	13 (65.0)	0.73
Perceived stress score	13.35 (4.68)	11.70 (6.04)	0.34
Mouthwash use, *n* (%)	7 (35.0)	13 (65.0)	0.11
Smoking status, *n* (%)			0.83
Never	10 (50.0)	11 (55.0)	
Former	3 (15.0)	4 (20.0)	
Current	7 (35.0)	5 (25.0)	
Alcohol consumption, g/day	1.54 (3.27)	3.87 (6.11)	0.14
Physical activity, MET-h/week	25.30 (32.39)	9.32 (12.64)	0.05
Body mass index, kg/m^2^	31.85 (4.73)	34.17 (4.49)	0.12
Insulin, µIU/mL	8.92 (5.08)	16.08 (10.55)	0.01
Glucose, mg/dL	86.00 (5.87)	96.30 (9.31)	<0.001
HbA1c, %	5.48 (0.24)	5.95 (0.28)	<0.001
HOMA-IR, mg/dL	1.93 (1.17)	3.90 (2.72)	0.01
Total Cholesterol, mg/dL	202.85 (39.38)	204.75 (42.73)	0.88
Triglyceride, mg/dL	131.80 (66.93)	204.75 (122.98)	0.03
HDL-C, mg/dL	46.55 (15.89)	40.80 (9.86)	0.18
LDL-C, mg/dL	129.95 (32.45)	122.95 (30.59)	0.49
C-reactive protein, mg/dL	3.73 (3.15)	5.91 (4.39)	0.08
IL-6, pg/mL	1.16 (1.25)	1.14 (0.55)	0.95
ICAM-1, ng/mL	660.55 (374.77)	597.98 (116.91)	0.60
VCAM-1, ng/mL	665.17 (165.11)	685.01 (208.80)	0.80
Adiponectin, μg/mL	11.07 (4.91)	7.76 (5.55)	0.14

Data are presented as frequency (percentage, %) and mean (standard deviation, SD) for categorical and continuous variables, respectively.

*p*-values were compared between cases and controls using χ^2^ tests and two-sample *t*-test for categorical and continuous variables, respectively. When frequency was <5 in cells, Fisher’s exact test was used for categorical variables.

HOMA-IR, Homeostatic Model Assessment for Insulin Resistance; HbA1c, glycosylated hemoglobin; HDL-C high-density lipoprotein cholesterol; IL-6, interleukin 6; ICAM-1, intercellular adhesion molecule-1; LDL, low-density lipoprotein; MET, metabolic equivalent; SD, standard deviation; SOALS, San Juan Overweight Adults Longitudinal Study; VCAM-1, vascular cellular adhesion molecule-1.

**Figure 1:**
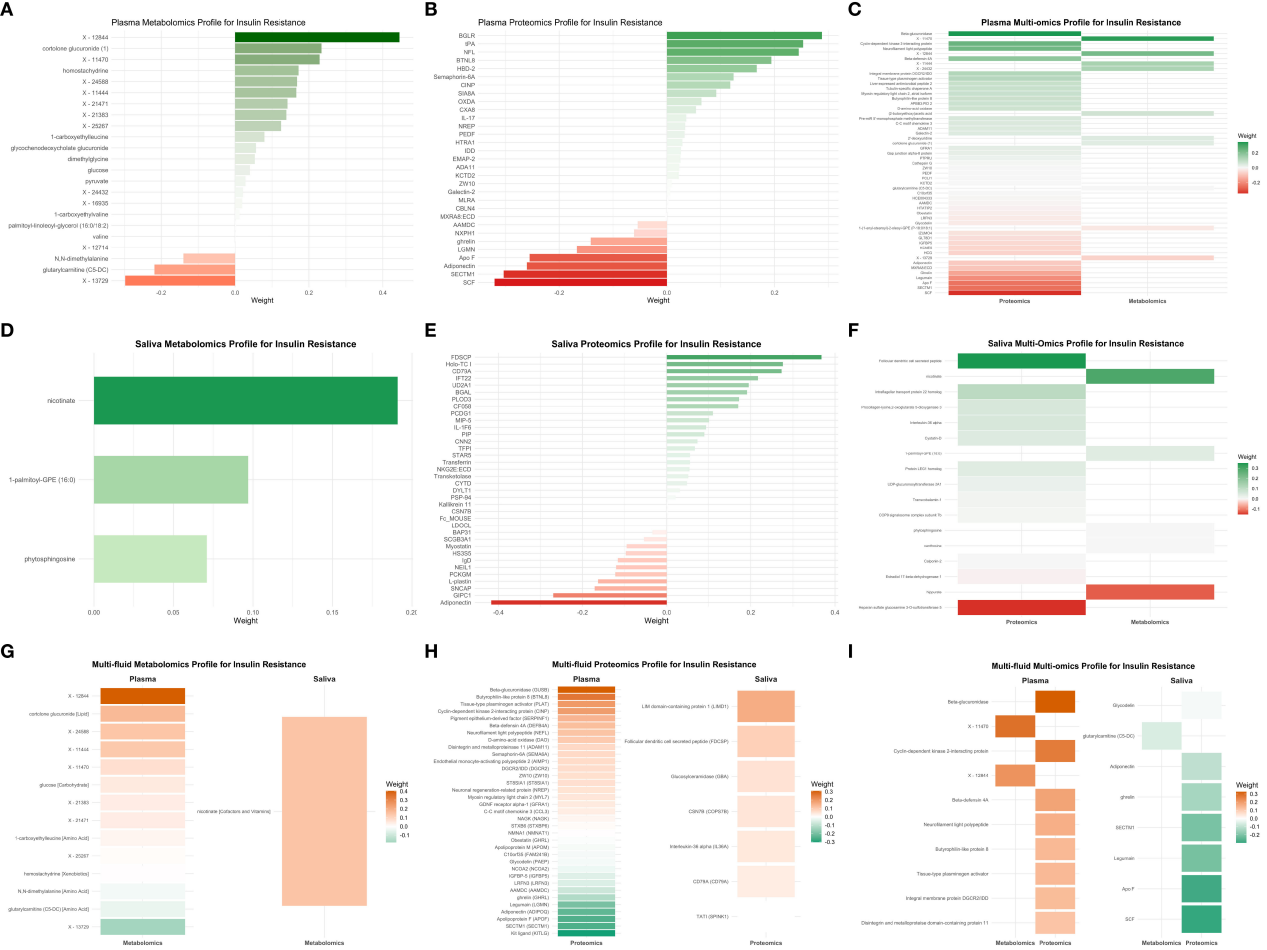
Plasma, saliva, multi-fluid metabolomics, proteomics, and multi-omics profile for insulin resistance. **(A)** Plasma metabolomics profile for insulin resistance. **(B)** Plasma proteomics profile for insulin resistance. **(C)** Plasma multi-omics profile for insulin resistance. **(D)** Saliva metabolomics profile for insulin resistance. **(E)** Saliva proteomics profile for insulin resistance. **(F)** Saliva multi-omics profile for insulin resistance. **(G)** Multi-fluid metabolomics profile for insulin resistance. **(H)** Multi-fluid proteomics profile for insulin resistance. **(I)** Multi-fluid multi-omics profile for insulin resistance.

Among the nine omics signatures evaluated, the multi-fluid multi-omics signature selected the largest number of features (*n* = 58), followed by the plasma multi-omics signature (*n* = 53), and the multi-fluid proteomics signature (*n* = 42). In contrast, the saliva metabolomics signature selected the fewest features (*n* = 3), followed by the saliva multi-omics signature (*n* = 17) (**Table 2**, **Figure 1**). Within the plasma metabolomics profile, cortolone glucuronide (weight = 0.234) and two unknown metabolites [X–12844 (weight = 0.445); X–11470 (weight = 0.229)] were identified as the top positively weighted metabolites, whereas unknown metabolite X–13729 (weight = −0.298), glutarylcarnitine (weight = −0.218), and *N*,*N*-dimethylalanine (weight = −0.140) were the top inversely weighted metabolites (**Figure 1a**, **Supplementary Table S1**). The plasma proteomics profile identified beta-glucuronidase (weight = 0.288), tissue-type plasminogen activator (weight = 0.253), and butyrophilin-like protein 8 (weight = 0.245) as the top positively weighted proteins, while kit ligand (KITLG) (weight = −0.320), secreted and transmembrane protein 1 (SECTM1) (weight = −0.303), and adiponectin (weight = −0.260) were the top inversely weighted proteins (**Figure 1b**, **Supplementary Table S2**). The plasma multi-omics profile combined these, with protein beta-glucuronidase (weight = 0.345), cyclin-dependent kinase 2-interacting (weight = 0.330), and unknown metabolite X–11470 (weight = 0.242) as positive contributors, and protein KITLG (weight = −0.343), SECTM1 (weight = −0.248), and apolipoprotein F (weight = −0.241) as the top inversely weighted features (**Figure 1c**, **Supplementary Table S3**).

**Table 2:** Associations of *omic* signatures of insulin resistance with incident T2D in SOALS.

Omic signatures	Number of features in signature	Model 1 HR	Model 1 95 %CI		Model 1 P	Model 2 HR	Model 2 95% CI		Model 2 P
Plasma metabolomics	23	1.39	0.96	2.02	0.08	1.23	0.70	2.17	0.47
Plasma proteomics	31	3.08	1.76	5.39	<0.001	3.13	1.08	9.10	0.04
Plasma multi-omics	53	2.74	1.50	5.00	0.001	3.68	1.27	10.63	0.02
Saliva metabolomics	3	0.16	0.04	0.67	0.01	0.04	0.002	0.68	0.03
Saliva proteomics	36	0.92	0.60	1.42	0.71	1.22	0.55	2.70	0.62
Saliva multi-omics	17	1.30	0.86	1.96	0.21	2.12	0.95	4.70	0.07
Multi-fluid metabolomics	15	1.31	0.89	1.94	0.17	1.05	0.69	1.61	0.82
Multi-fluid proteomics	42	3.04	1.68	5.50	<0.001	3.14	0.94	10.46	0.06
Multi-fluid multi-omics	58	2.58	1.41	4.74	0.002	3.25	1.08	9.81	0.04

Conditional logistic regression was used to evaluate associations between omics-derived signatures and incident type 2 diabetes. Hazard ratio, 95% CI, and *p*-values are reported.

Model 1 was a crude conditional model that accounted for the matching factors (age and sex) by design but included no additional covariates. Model 2 additionally adjusted for potential confounders using an outcome-free, low-dimensional confounder score. Specifically, we constructed a principal component (PC)-based score derived from the following variables: smoking status, income, education, psychosocial stress, physical activity, alcohol consumption, mouthwash use, family history of diabetes, and body mass index. We summarized potential confounders into outcome-free PC scores computed from the covariate matrix (numeric variables standardized; indicator variables unscaled). To balance confounding control with numerical stability, we prespecified adjustment for PC1–PC4 as a parsimonious set.

CI, confidence interval; HR, hazard ratio; SOALS, San Juan Overweight Adults Longitudinal Study.

The saliva metabolomics profile only included nicotinate (weight = 0.191), phytosphingosine (weight = 0.097), and 1-palmitoyl-GPE (16:0) (weight = 0.071) as positive features (**Figure 1d**, **Supplementary Table S4**). In the saliva proteomics profile, follicular dendritic cell secreted peptide (weight = 0.368), transcobalamin-1 (weight = 0.276), and B-cell antigen receptor complex-associated protein alpha chain (weight = 0.273) were identified as the top positive weighted proteins. On the other hand, adiponectin (weight = −0.417), PDZ domain-containing protein GIPC1 (weight = −0.270), and synphilin-1 (weight = −0.171) were the top inversely weighted proteins (**Figure 1e**, **Supplementary Table S5**). The saliva multi-omics profile combined these, with protein follicular dendritic cell secreted peptide (weight = 0.350) and metabolite nicotinate (weight = 0.291) as shared positive features, and protein heparan sulfate glucosamine 3-O-sulfotransferase 5 (weight = −0.153) and metabolite hippurate (weight = −0.125) as unique inverse contributors (**Figure 1f**, **Supplementary Table S6**).

The multi-fluid metabolomics profile highlighted unknown metabolite X–12844 (weight = 0.404), cortolone glucuronide (weight = 0.171), and nicotinate (weight = 0.144) as positively weighted metabolites. Nicotinate overlapped with saliva metabolomics profile, while cortolone glucuronide and unknown metabolite X–12844 were unique to the plasma metabolomics signature. Inversely weighted features included unknown metabolite X–13729 (weight = −0.179), glutarylcarnitine (weight = −0.041), and *N*,*N*-dimethylalanine (weight = −0.025), all shared with plasma metabolomics (**Figure 1g**, **Supplementary Table S7**). The multi-fluid proteomics profile identified beta-glucuronidase (weight = 0.308), butyrophilin-like protein 8 (weight = 0.258), and tissue-type plasminogen activator (weight = 0.192) as top positively weighted proteins, all of which also appeared in the plasma proteomics profile. Conversely, KITLG (weight = −0.302), SECTM1 (weight = −0.253), apolipoprotein F (weight = −0.250), and adiponectin (weight = −0.231) were among the most strongly negatively weighted proteins, consistent with findings from plasma proteomics. Adiponectin, one of the top inversely weighted proteins for multi-fluid proteomics profile, was consistently identified in both plasma and saliva proteomics profiles (**Figure 1h**, **Supplementary Table S8**). Finally, the multi-fluid multi-omics profile combined features from plasma and saliva, with protein beta-glucuronidase (weight = 0.326), cyclin-dependent kinase 2-interacting protein (weight = 0.266), and unknown metabolite X–1147 (weight = 0.288), consistent with plasma multi-omics, as top positive contributors. Inversely top weighted features included protein KITLG (weight = −0.297), apolipoprotein F (weight = −0.283), and legumain (weight = −0.213), all derived from plasma multi-omics (**Figure 1i**, **Supplementary Table S9**).

**Figure 2:**
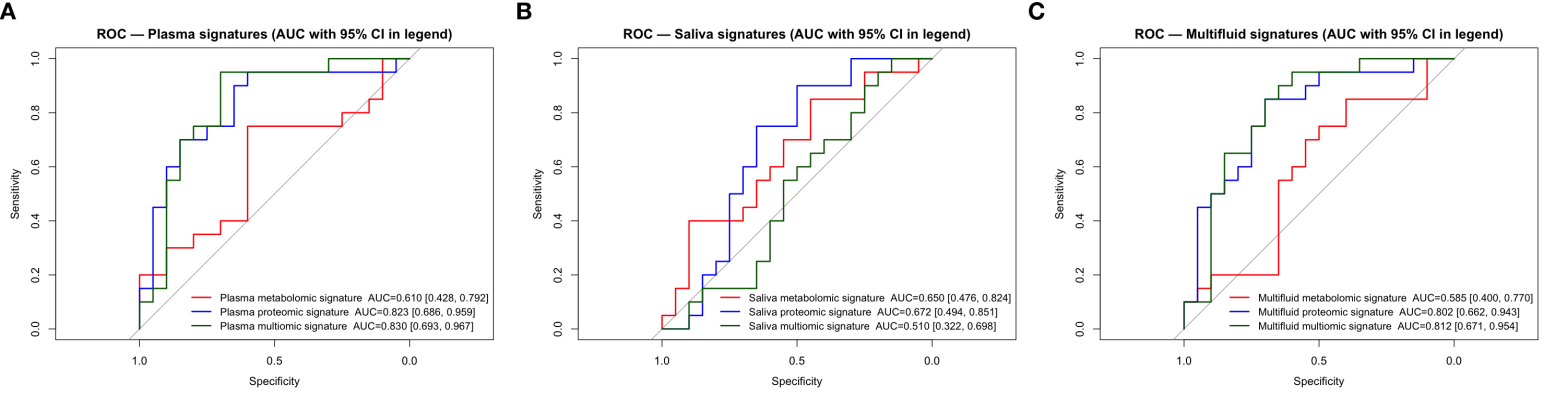
ROC comparison for T2D across omics signatures. **(A)** Comparison of ROC curves for plasma signatures. **(B)**. Comparison of ROC curves for saliva signatures. **(C)** Comparison of ROC curves for multi-fluid signatures. ROC, receiver operating characteristic; AUC, area under the curve; T2D, type 2 diabetes.

The plasma multi-omics (AUC = 0.83; 95% CI: 0.67–0.96), plasma proteomics (AUC = 0.82; 95% CI: 0.68–0.94), multi-fluid multi-omics (AUC = 0.81; 95% CI: 0.65–0.95), and multi-fluid proteomics (AUC = 0.80; 95% CI: 0.66–0.93) showed the strongest discrimination with significant permutation *p*-values (≤0.003). Saliva-based signatures and metabolomics-only models had limited performance (AUCs = 0.51–0.67) and were not significant (**Figures 2**, **Supplementary Table S10**). Bootstrap 95% CIs, conditional on the fixed LOOCV scores, were narrow and centered around the observed AUCs, supporting stability under our validation workflow.

For the outcome-free PC-based confounder score, the first three PCs explained 60.4% of the covariate variance, and the first four explained 72.7%. The 80% variance threshold required five PCs, but adding a fifth PC produced numerical instability in matched risk-set models, yielding inflated and imprecise HR estimates, consistent with limited within-risk-set variation and few discordant sets given our small sample (*N* = 40). To balance confounding control with numerical stability, we prespecified adjustment for PC1–PC4 as a parsimonious set. In the multivariable-adjusted Model 2, each SD increase in plasma proteomics (HR = 3.13, 95% CI: 1.08–9.10), plasma multi-omics (HR = 3.68, 95% CI: 1.27–10.63), and multi-fluid multi-omics (HR = 3.25, 95% CI: 1.08–9.81) signature was associated with a threefold higher risk of incident T2D. Saliva metabolomics signature was inversely associated with T2D (HR = 0.04, 95% CI: 0.002–0.68). Neither the plasma metabolomics signature nor saliva proteomics signatures were significantly associated with T2D risk in multivariable-adjusted Model 2 (**Table 2**).

## Discussion

In this study, comprehensive profiling of 1,051 plasma metabolites, 635 saliva metabolites, and 7,595 proteins across plasma and saliva samples identified nine distinct omics signatures reflective of baseline insulin resistance. Among these, plasma proteomics, multi-omics, and multi-fluid proteomics and multi-omics signatures exhibited the strongest discriminatory ability for incident T2D. In contrast, saliva-based omics and plasma metabolomics demonstrated modest predictive performance, and their integration into plasma-based predictive models did not improve discrimination. Predictive performance of T2D from multi-fluid signatures was largely driven by plasma-derived omics. These findings underscore the superior utility of plasma proteomic data for predicting T2D in this high-risk Puerto Rican population.

Consistent with findings from prior studies in the UK (20, 21), the US (white persons and black persons) (11), and China (22), we found plasma proteomic signatures to be significantly associated with incident T2D. Importantly, this study is the first to validate these associations in Puerto Rican adults, a historically underrepresented group in T2D and omics research. Contrary to our previous work in the SOALS cohort (8) among 849 participants where plasma metabolomic signatures reported higher predictive accuracy (AUC = 0.75), the current pilot showed only modest predictive accuracy (AUC = 0.61), and plasma metabolomic signatures of HOMA-IR were not significantly associated with incident T2D. These differences may reflect the smaller sample size and analytic approach used in the present pilot. Although adding plasma metabolomics to plasma proteomics (AUC = 0.82) did not improve overall T2D prediction (combined AUC = 0.83), it strengthened the association between this molecular profile and T2D. The larger HRs with the plasma multi-omics profile suggest that metabolomics may provide complementary etiological information and enhance biological interpretability even if it did not contribute additional predictive power in this small pilot study. This suggests that larger studies integrating both plasma metabolomics and proteomics for T2D prediction are warranted to explore this further.

Saliva-based omics profiles, while minimally invasive and promising in theory, demonstrated limited utility in this pilot study. The saliva metabolomics signature was inversely associated with T2D, which needs to be interpreted cautiously. Saliva metabolites are strongly influenced by oral-microbiome activity (e.g., microbial metabolism and host–microbe co-metabolites), which may index a more favorable oral ecological state linked to lower systemic inflammation and improved metabolic health. Notably, in our dataset, the saliva signature showed limited prediction for T2D, suggesting that it may primarily reflect oral-microbiome-related processes rather than T2D. Because we did not measure the saliva microbiome, we could not directly test this mechanism; future studies integrating paired salivary microbiome with metabolomics will be essential to validate these pathways and clarify causal links to T2D risk. Neither salivary proteomics nor multi-omics signatures were significantly associated with incident T2D. The modest predictive ability of saliva metabolomics (AUC = 0.65) is consistent with previous findings from the larger SOALS cohort, where a saliva metabolomic signature of insulin resistance was associated with incident prediabetes but not T2D (AUC = 0.69) (8). In contrast, a prior study involving 2,974 Qatari participants and 1,317 salivary proteins reported modestly better predictive performance for T2D (AUC = 0.76), suggesting that differences in sample size and population characteristics may account for the discrepancies between findings (13).

Multi-fluid signatures were largely influenced by plasma-derived omics, particularly for multi-fluid proteomics and multi-fluid multi-omics signatures, suggesting that the predictive capability of these integrated profiles may be driven by plasma-omics data rather than saliva-omics data.

Within the plasma proteome, several top-ranked proteins were consistent with established biological mechanisms and prior large-cohort findings. β-glucuronidase (BGLR), a lysosomal enzyme that hydrolyzes glucuronides and promotes deconjugation of xenobiotics and hormones, can increase oxidative stress and inflammation when released extracellularly (9). Elevated circulating levels have been linked to hepatic and metabolic dysfunction and to higher T2D risk in the EPIC-Norfolk study (9). Tissue-type plasminogen activator (tPA), an endothelial-derived serine protease involved in fibrinolysis, reflects endothelial dysfunction and chronic inflammation; higher levels were associated with insulin resistance in the Middle-Aged Soweto Cohort (10), which is consistent with our findings. In contrast, adiponectin, an adipokine that enhances insulin sensitivity and fatty acid oxidation, was inversely weighted in our signature. Its protective role has been confirmed in a meta-analysis of 13 prospective studies (involving >14,000 participants) (23), showing a 28% lower T2D risk per log-μg/mL increase. These findings were also corroborated by ARIC (11) and UK Biobank (24) findings. Together, these consistent biological functions and directionality across large studies and diverse populations reinforce the mechanistic and epidemiologic plausibility of our proteomic signature. Other inversely weighted novel proteins such as SECTM1 and KITLG may reflect anti-inflammatory or immunoregulatory activity that protects against metabolic dysfunction (25, 26). While their roles in T2D remain understudied, these findings suggest new candidates for mechanistic exploration.

For plasma metabolomics, cortolone glucuronide exhibited a strong positive weight in relation to T2D, consistent with findings from the SOALS study (8). While prior research in Sweden has shown that lower circulating cortisol levels are associated with higher T2D risk, this may reflect increased cortisol clearance (6). The elevated cortolone glucuronide observed here could indicate enhanced metabolic conversion of cortisol, pointing to a compensatory pathway linking cortisol metabolism to T2D pathophysiology. The top weighted metabolites in the plasma metabolomic profile predominantly reflect perturbations in amino acid metabolism (particularly branched-chain amino acids like leucine, isoleucine, and valine), lipid-related pathways (including corticosteroids and bile acids), and central energy metabolism (glycolysis and pyruvate metabolism), all pathways that have been highlighted in previous investigations of associations between metabolites and T2D among Puerto Rican adults (27–29). This suggests a shared pathway architecture involving glucocorticoid activity, insulin resistance, and mitochondrial energy utilization, which are central to T2D pathophysiology.

Salivary proteomic signatures implicated several immune-related proteins, such as transcobalamin-1 and cystatin-D, as biomarkers for T2D consistent with previous studies (14). We also identified a novel protein, follicular dendritic cell-secreted peptide (FDC-SP), which was positively associated with T2D risk. FDC-SP is involved in B-cell activation and IgA regulation and is primarily known for its role in mucosal immunity (30). Although FDC-SP has not previously been linked to insulin resistance or T2D, its immunomodulatory properties suggest a potential role in immune-metabolic regulation (30) and further investigation is warranted to elucidate this relationship. Salivary adiponectin levels have been inversely associated with T2D in our study, consistent with previous research. In India, newly diagnosed patients with T2D exhibited significantly reduced salivary adiponectin compared to healthy controls (31). Similarly, in individuals with impaired glucose tolerance, lower baseline plasma adiponectin levels predicted progression to T2D (11.3 ± 5.5 *vs*. 16.7 ± 7.6 μg/mL, *p* = 0.0017) (32). In our study, we also observed decreased salivary levels of PDZ domain-containing protein GIPC1 and Synphilin-1 in individuals with insulin resistance and T2D, which may reflect impaired lipid handling (33) and energy homeostasis (34), but these findings require validation in larger studies. We identified only three salivary metabolites, nicotinate, 1-palmitoyl-GPE (16:0), and phytosphingosine. Nicotinate, in particular, may reflect smoking-related metabolic responses. The multi-fluid metabolomic score for insulin resistance was driven primarily by plasma metabolites, and the multi-fluid multi-omics signature was largely shaped by plasma-based multi-omics, suggesting that plasma has greater information for predicting T2D risk compared to saliva. Replication and refinement of these findings are needed to further elucidate the role of saliva in understanding T2D pathophysiology and enhancing its prediction.

Our study has several notable strengths. To our knowledge, it is the first to comprehensively investigate combined saliva-based and plasma-based metabolomics and proteomics, as well as multi-fluid, multi-omics profiles, in relation to T2D in Puerto Ricans, an underrepresented and high-risk population with established health disparities. The study design incorporated repeated assessments of T2D biomarkers, extensive covariate data, and comprehensive profiling of a large number of metabolites and proteins. Nonetheless, our findings should be interpreted considering several limitations. The small sample size and the large number of *omic* features reduce statistical power and limit generalizability. However, we leveraged machine learning-based data reduction approaches combined with several types of cross-validation, bootstrap, and permutation test to minimize overfitting and ensure result stability. Selection bias is possible, as participants were volunteers in the SOALS study and may represent a healthier or more health-conscious subset of Puerto Rican adults. Information bias could also arise from measurement error in fasting status or other covariates, although biospecimens were collected under standardized protocols. The observational nature of the study precludes causal inference, and residual confounding cannot be excluded. Furthermore, metabolomic and proteomic profiling were restricted to baseline measurements, which prevented the analysis of temporal changes in metabolite and protein levels. In addition, several top metabolite features remain uncharacterized, highlighting the need for future annotation to improve biological interpretation. Finally, because this study included only Puerto Rican adults and in the absence of external validation data with multi-fluid multi-omics, the findings may not be broadly applicable to other Hispanic groups or other populations. Accordingly, these promising findings are exploratory and require confirmation in larger, independent, and more diverse cohorts.

## Conclusion

In summary, we identified plasma proteomics and multi-omics signatures, as well as multi-fluid metabolomics, multi-fluid proteomics, and multi-fluid multi-omics signatures, predictive of insulin resistance and associated with incident T2D in Puerto Rican adults with overweight and obesity. Plasma proteomics demonstrated the strongest predictive value, while saliva-based profiles offered only moderate utility and did not enhance discrimination when combined with plasma data. The predictive performance for T2D based on multi-fluid signatures was primarily driven by plasma-derived proteomics. Future research should focus on elucidating the biological roles of these metabolites and proteins in T2D pathophysiology and assessing the integration of multi-omics profiling with traditional risk factors for early detection and prevention of T2D.

## Data Availability

The raw data supporting the conclusions of this article will be made available by the authors, without undue reservation. Data inquiries can be directed to the corresponding author.
