# Supplementary material for: Multi-fluid, multi-omics signatures of insulin resistance and incident type 2 diabetes among Puerto Rican adults
Source: Front Endocrinol (Lausanne). 2025 Dec 4;16:1699656. doi: 10.3389/fendo.2025.1699656 (PMC12711532; doi:10.3389/fendo.2025.1699656)
Supplement: Supplementary file 1 [file Table1.docx]

**Supplement Table 1**. Plasma metabolomics profile for insulin resistance

| Metabolite Name | Super Pathway | Sub-pathway | Weight in signature |
| --- | --- | --- | --- |
| X - 12844 |  |  | 0.445 |
| cortolone glucuronide (1) | Lipid | Corticosteroids | 0.234 |
| X - 11470 |  |  | 0.229 |
| homostachydrine | Xenobiotics | Food Component/Plant | 0.172 |
| X - 24588 |  |  | 0.168 |
| X - 11444 |  |  | 0.166 |
| X - 21471 |  |  | 0.142 |
| X - 21383 |  |  | 0.139 |
| X - 25267 |  |  | 0.125 |
| 1-carboxyethylleucine | Amino Acid | Leucine, Isoleucine and Valine Metabolism | 0.079 |
| glycochenodeoxycholate glucuronide | Lipid | Primary Bile Acid Metabolism | 0.056 |
| dimethylglycine | Amino Acid | Glycine, Serine and Threonine Metabolism | 0.053 |
| glucose | Carbohydrate | Glycolysis, Gluconeogenesis, and Pyruvate Metabolism | 0.040 |
| pyruvate | Carbohydrate | Glycolysis, Gluconeogenesis, and Pyruvate Metabolism | 0.028 |
| X - 24432 |  |  | 0.021 |
| X - 16935 |  |  | 0.019 |
| 1-carboxyethylvaline | Amino Acid | Leucine, Isoleucine and Valine Metabolism | 0.012 |
| palmitoyl-linoleoyl-glycerol (16:0/18:2) | Lipid | Diacylglycerol | 0.003 |
| valine | Amino Acid | Leucine, Isoleucine and Valine Metabolism | 0.001 |
| X - 12714 |  |  | -0.001 |
| N,N-dimethylalanine | Amino Acid | Alanine and Aspartate Metabolism | -0.140 |
| glutarylcarnitine (C5-DC) | Amino Acid | Lysine Metabolism | -0.218 |
| X - 13729 |  |  | -0.298 |
| All metabolites beginning with “X –“ are unknown metabolites. The number assigned to each unknown was provided by Metabolon, Inc. | | | |

**Supplement Table 2**. Plasma proteomics profile for insulin resistance

| Protein Name | Reported Name or Label | Entrez Gene Symbol | Weight in signature |
| --- | --- | --- | --- |
| Beta-glucuronidase | BGLR | GUSB | 0.288 |
| Tissue-type plasminogen activator | tPA | PLAT | 0.253 |
| Neurofilament light polypeptide | NFL | NEFL | 0.245 |
| Butyrophilin-like protein 8 | BTNL8 | BTNL8 | 0.194 |
| Beta-defensin 4A | HBD-2 | DEFB4A | 0.167 |
| Semaphorin-6A | Semaphorin-6A | SEMA6A | 0.124 |
| Cyclin-dependent kinase 2-interacting protein | CINP | CINP | 0.118 |
| Alpha-N-acetylneuraminide alpha-2,8-sialyltransferase | SIA8A | ST8SIA1 | 0.092 |
| D-amino-acid oxidase | OXDA | DAO | 0.064 |
| Gap junction alpha-8 protein | CXA8 | GJA8 | 0.054 |
| Interleukin-17A | IL-17 | IL17A | 0.036 |
| Neuronal regeneration-related protein | NREP | NREP | 0.034 |
| Pigment epithelium-derived factor | PEDF | SERPINF1 | 0.033 |
| Serine protease HTRA1 | HTRA1 | HTRA1 | 0.029 |
| Integral membrane protein DGCR2/IDD | IDD | DGCR2 | 0.026 |
| Endothelial monocyte-activating polypeptide 2 | EMAP-2 | AIMP1 | 0.025 |
| Disintegrin and metalloproteinase domain-containing protein 11 | ADA11 | ADAM11 | 0.023 |
| BTB/POZ domain-containing protein KCTD2 | KCTD2 | KCTD2 | 0.022 |
| Centromere/kinetochore protein zw10 homolog | ZW10 | ZW10 | 0.009 |
| Galectin-2 | Galectin-2 | LGALS2 | 0.006 |
| Myosin regulatory light chain 2, atrial isoform | MLRA | MYL7 | 0.003 |
| Cerebellin-4 | CBLN4 | CBLN4 | 0.001 |
| Matrix-remodeling-associated protein 8:Extracellular domain | MXRA8:ECD | MXRA8 | -0.003 |
| Mth938 domain-containing protein | AAMDC | AAMDC | -0.054 |
| Neurexophilin-1 | NXPH1 | NXPH1 | -0.061 |
| Appetite-regulating hormone | ghrelin | GHRL | -0.141 |
| Legumain | LGMN | LGMN | -0.167 |
| Apolipoprotein F | Apo F | APOF | -0.255 |
| Adiponectin | Adiponectin | ADIPOQ | -0.260 |
| Secreted and transmembrane protein 1 | SECTM1 | SECTM1 | -0.303 |
| Kit ligand | SCF | KITLG | -0.320 |

**Supplement Table 3**. Plasma multi-omics profile for insulin resistance

| Protein | | | Metabolite | | | Weight in signature |
| --- | --- | --- | --- | --- | --- | --- |
| Protein Name | Reported Name or Label | Entrez Gene Symbol | Metabolite Name | Super Pathway | Sub-pathway |  |
| Beta-glucuronidase | BGLR | GUSB |  |  |  | 0.345 |
|  |  |  | X - 11470 |  |  | 0.330 |
| Cyclin-dependent kise 2-interacting protein | CINP | CINP |  |  |  | 0.242 |
| Neurofilament light polypeptide | NFL | NEFL |  |  |  | 0.235 |
|  |  |  | X - 12844 |  |  | 0.223 |
| Beta-defensin 4A | HBD-2 | DEFB4A |  |  |  | 0.182 |
|  |  |  | X - 11444 |  |  | 0.138 |
|  |  |  | X - 24432 |  |  | 0.133 |
| Integral membrane protein DGCR2/IDD | IDD | DGCR2 |  |  |  | 0.121 |
| Tissue-type plasminogen activator | tPA | PLAT |  |  |  | 0.115 |
| Liver-expressed antimicrobial peptide 2 | LEAP2 | LEAP2 |  |  |  | 0.087 |
| Tubulin-specific chaperone A | TBCA | TBCA |  |  |  | 0.085 |
| Myosin regulatory light chain 2, atrial isoform | MLRA | MYL7 |  |  |  | 0.083 |
| Butyrophilin-like protein 8 | BTNL8 | BTNL8 |  |  |  | 0.080 |
| Amyloid beta A4 precursor protein-binding family B member 3:Phosphotyrosine Interaction Domain 2, Isoform IV | APBB3:PID 2 | APBB3 |  |  |  | 0.079 |
| D-amino-acid oxidase | OXDA | DAO |  |  |  | 0.075 |
|  |  |  | (2-butoxyethoxy)acetic acid | Xenobiotics | Chemical | 0.069 |
| Pre-miR 5'-monophosphate methyltransferase | BN3D2 | BCDIN3D |  |  |  | 0.056 |
| C-C motif chemokine 3 | MIP-1a | CCL3 |  |  |  | 0.052 |
| Disintegrin and metalloproteise domain-containing protein 11 | ADA11 | ADAM11 |  |  |  | 0.048 |
| Galectin-2 | Galectin-2 | LGALS2 |  |  |  | 0.046 |
|  |  |  | 2'-deoxyuridine | Nucleotide | Pyrimidine Metabolism, Uracil containing | 0.046 |
|  |  |  | cortolone glucuronide (1) | Lipid | Corticosteroids | 0.041 |
| GDNF family receptor alpha-1 | GFRa-1 | GFRA1 |  |  |  | 0.036 |
| Gap junction alpha-8 protein | CXA8 | GJA8 |  |  |  | 0.031 |
| Receptor-type tyrosine-protein phosphatase U | PTPRU | PTPRU |  |  |  | 0.019 |
| Cathepsin G | Cathepsin G | CTSG |  |  |  | 0.005 |
| Centromere/kinetochore protein zw10 homolog | ZW10 | ZW10 |  |  |  | 0.004 |
| Pigment epithelium-derived factor | PEDF | SERPINF1 |  |  |  | 0.004 |
| PTB-containing, cubilin and LRP1-interacting protein | PCLI1 | PID1 |  |  |  | 0.002 |
| BTB/POZ domain-containing protein KCTD2 | KCTD2 | KCTD2 |  |  |  | 0.0001 |
|  |  |  | glutarylcarnitine (C5-DC) | Amino Acid | Lysine Metabolism | -0.002 |
| Uncharacterized protein C10orf35 | CJ035 | FAM241B |  |  |  | -0.002 |
| HCE004333 | HCE004333 | HCE004333 |  |  |  | -0.004 |
| Mth938 domain-containing protein | AAMDC | AAMDC |  |  |  | -0.006 |
| Oxidoreductase HTATIP2 | HTAI2 | HTATIP2 |  |  |  | -0.011 |
| Obestatin | Obestatin | GHRL |  |  |  | -0.019 |
| Glycodelin | Glycodelin | PAEP |  |  |  | -0.023 |
| Leucine-rich repeat and fibronectin type-III domain-containing protein 3 | LRFN3 | LRFN3 |  |  |  | -0.023 |
|  |  |  | 1-(1-enyl-stearoyl)-2-oleoyl-GPE (P-18:0/18:1) | Lipid | Plasmalogen | -0.030 |
| Izumo sperm-egg fusion protein 4 | IZUM4 | IZUMO4 |  |  |  | -0.038 |
| Glycosyltransferase 8 domain-containing protein 1 | GL8D1 | GLT8D1 |  |  |  | -0.046 |
| Insulin-like growth factor-binding protein 5 | IGFBP-5 | IGFBP5 |  |  |  | -0.056 |
| Potassium voltage-gated channel subfamily E regulatory beta subunit 5:Cytoplasmic domain | KCE1L:CD | KCNE5 |  |  |  | -0.062 |
| Human Chorionic Godotropin | HCG | CGA\|CGB3\|CGB7 | |  |  | -0.065 |
|  |  |  | X - 13729 |  |  | -0.069 |
| Adiponectin | Adiponectin | ADIPOQ |  |  |  | -0.086 |
| Matrix-remodeling-associated protein 8:Extracellular domain | MXRA8:ECD | MXRA8 |  |  |  | -0.096 |
| Appetite-regulating hormone | ghrelin | GHRL |  |  |  | -0.155 |
| Legumain | LGMN | LGMN |  |  |  | -0.195 |
| Apolipoprotein F | Apo F | APOF |  |  |  | -0.241 |
| Secreted and transmembrane protein 1 | SECTM1 | SECTM1 |  |  |  | -0.248 |
| Kit ligand | SCF | KITLG |  |  |  | -0.343 |
| All metabolites beginning with “X –“ are unknown metabolites. The number assigned to each unknown was provided by Metabolon, Inc. | | | | | | |

**Supplement Table 4**. Saliva metabolomics profile for insulin resistance

| Metabolite Name | Super Pathway | Sub-pathway | Weight in signature |
| --- | --- | --- | --- |
| nicotinate | Cofactors and Vitamins | Nicotinate and Nicotinamide Metabolism | 0.191 |
| 1-palmitoyl-GPE (16:0) | Lipid | Lysophospholipid | 0.097 |
| phytosphingosine | Lipid | Sphingolipid Synthesis | 0.071 |

**Supplement Table 5.** Saliva proteomics profile for insulin resistance

| Protein Name | Reported Name or Label | EntrezGeneSymbol | Weight in signature |
| --- | --- | --- | --- |
| Follicular dendritic cell secreted peptide | FDSCP | FDCSP | 0.368 |
| Transcobalamin-1 | Holo-TC I | TCN1 | 0.276 |
| B-cell antigen receptor complex-associated protein alpha chain | CD79A | CD79A | 0.273 |
| Intraflagellar transport protein 22 homolog | IFT22 | IFT22 | 0.217 |
| UDP-glucuronosyltransferase 2A1 | UD2A1 | UGT2A1 | 0.195 |
| Beta-galactosidase | BGAL | GLB1 | 0.191 |
| Procollagen-lysine,2-oxoglutarate 5-dioxygenase 3 | PLOD3 | PLOD3 | 0.172 |
| Protein LEG1 homolog | CF058 | LEG1 | 0.170 |
| Protocadherin gamma-A1 | PCDG1 | PCDHGA1 | 0.110 |
| C-C motif chemokine 15 | MIP-5 | CCL15 | 0.101 |
| Interleukin-36 alpha | IL-1F6 | IL36A | 0.094 |
| Prolactin-inducible protein | PIP | PIP | 0.089 |
| Calponin-2 | CNN2 | CNN2 | 0.073 |
| Tissue factor pathway inhibitor | TFPI | TFPI | 0.067 |
| Serotransferrin | Transferrin | TF | 0.055 |
| StAR-related lipid transfer protein 5 | STAR5 | STARD5 | 0.055 |
| NKG2-E type II integral membrane protein:Isoform E, Extracellular domain | NKG2E:ECD | KLRC3 | 0.054 |
| Transketolase | Transketolase | TKT | 0.051 |
| Cystatin-D | CYTD | CST5 | 0.048 |
| Dynein light chain Tctex-type 1 | DYLT1 | DYNLT1 | 0.031 |
| Beta-microseminoprotein | PSP-94 | MSMB | 0.021 |
| COP9 signalosome complex subunit 7b | CSN7B | COPS7B | 0.002 |
| Kallikrein-11 | Kallikrein 11 | KLK11 | 0.002 |
| Fc_MOUSE | Fc_MOUSE |  | 0.00001 |
| Protein LDOC1L | LDOCL | RTL6 | -0.0001 |
| B-cell receptor-associated protein 31 | BAP31 | BCAP31 | -0.034 |
| Secretoglobin family 3A member 1 | Secretoglobin family 3A member 1 | SCGB3A1 | -0.054 |
| Growth/differentiation factor 8 | Myostatin | MSTN | -0.095 |
| Heparan sulfate glucosamine 3-O-sulfotransferase 5 | HS3S5 | HS3ST5 | -0.097 |
| Immunoglobulin D | IgD | IGHD | -0.116 |
| Endonuclease 8-like 1 | NEIL1 | NEIL1 | -0.120 |
| Phosphoenolpyruvate carboxykinase [GTP], mitochondrial | PCKGM | PCK2 | -0.122 |
| Plastin-2 | L-plastin | LCP1 | -0.163 |
| Synphilin-1 | SNCAP | SNCAIP | -0.171 |
| PDZ domain-containing protein GIPC1 | GIPC1 | GIPC1 | -0.270 |
| Adiponectin | Adiponectin | ADIPOQ | -0.417 |

**Supplement Table 6**. Saliva multi-omics profile for insulin resistance

| Protein | | | Metabolite | | | Weight in signature |
| --- | --- | --- | --- | --- | --- | --- |
| Protein Name | Reported Name or Label | Entrez Gene Symbol | Metabolite Name | Super Pathway | Sub-pathway |  |
| Follicular dendritic cell secreted peptide | FDSCP | FDCSP |  |  |  | 0.350 |
|  |  |  | nicotinate | Cofactors and Vitamins | Nicotinate and Nicotinamide Metabolism | 0.291 |
| Intraflagellar transport protein 22 homolog | IFT22 | IFT22 |  |  |  | 0.106 |
| Interleukin-36 alpha | IL-1F6 | IL36A |  |  |  | 0.057 |
| Procollagen-lysine,2-oxoglutarate 5-dioxygenase 3 | PLOD3 | PLOD3 |  |  |  | 0.057 |
| Cystatin-D | CYTD | CST5 |  |  |  | 0.048 |
|  |  |  | 1-palmitoyl-GPE (16:0) | Lipid | Lysophospholipid | 0.042 |
| Protein LEG1 homolog | CF058 | LEG1 |  |  |  | 0.041 |
| UDP-glucuronosyltransferase 2A1 | UD2A1 | UGT2A1 |  |  |  | 0.022 |
| Transcobalamin-1 | Holo-TC I | TCN1 |  |  |  | 0.013 |
| COP9 signalosome complex subunit 7b | CSN7B | COPS7B |  |  |  | 0.007 |
|  |  |  | phytosphingosine | Lipid | Sphingolipid Synthesis | 0.004 |
|  |  |  | xanthosine | Nucleotide | Purine Metabolism, (Hypo)Xanthine/Inosine containing | 0.002 |
| Calponin-2 | CNN2 | CNN2 |  |  |  | 0.002 |
| Estradiol 17-beta-dehydrogenase 1 | 17-beta-HSD 1 | HSD17B1 |  |  |  | -0.005 |
|  |  |  | hippurate | Xenobiotics | Benzoate Metabolism | -0.125 |
| Heparan sulfate glucosamine 3-O-sulfotransferase 5 | HS3S5 | HS3ST5 |  |  |  | -0.153 |

**Supplement Table 7**. Multi-fluid metabolomics profile for insulin resistance

| Fluid Type | Metabolite Name | Super Pathway | Sub-pathway | Weight in signature |
| --- | --- | --- | --- | --- |
| Plasma | X - 12844 |  |  | 0.404 |
| Plasma | cortolone glucuronide | Lipid | Corticosteroids | 0.171 |
| Saliva | nicotinate | Cofactors and Vitamins | Nicotinate and Nicotinamide Metabolism | 0.144 |
| Plasma | X - 24588 |  |  | 0.142 |
| Plasma | X - 11444 |  |  | 0.135 |
| Plasma | X - 11470 |  |  | 0.075 |
| Plasma | glucose | Carbohydrate | Glycolysis, Gluconeogenesis, and Pyruvate Metabolism | 0.051 |
| Plasma | X - 21383 |  |  | 0.042 |
| Plasma | X - 21471 |  |  | 0.041 |
| Plasma | 1-carboxyethylleucine | Amino Acid | Leucine, Isoleucine and Valine Metabolism | 0.024 |
| Plasma | X - 25267 |  |  | 0.008 |
| Plasma | homostachydrine | Xenobiotics | Food Component/Plant | 0.003 |
| Plasma | N,N-dimethylalanine | Amino Acid | Alanine and Aspartate Metabolism | -0.025 |
| Plasma | glutarylcarnitine (C5-DC) | Amino Acid | Lysine Metabolism | -0.041 |
| Plasma | X - 13729 |  |  | -0.179 |
| All metabolites beginning with “X –“ are unknown metabolites. The number assigned to each unknown was provided by Metabolon, Inc. | | | | |

**Supplement Table 8**. Multi-fluid proteomics profile for insulin resistance

| Fluid Type | Protein Name | Reported Name or Label | EntrezGeneSymbol | Weight in signature |
| --- | --- | --- | --- | --- |
| Plasma | Beta-glucuronidase | BGLR | GUSB | 0.308 |
| Plasma | Butyrophilin-like protein 8 | BTNL8 | BTNL8 | 0.258 |
| Plasma | Tissue-type plasminogen activator | tPA | PLAT | 0.192 |
| Plasma | Cyclin-dependent kinase 2-interacting protein | CINP | CINP | 0.185 |
| Saliva | LIM domain-containing protein 1 | LIMD1 | LIMD1 | 0.155 |
| Plasma | Pigment epithelium-derived factor | PEDF | SERPINF1 | 0.140 |
| Plasma | Beta-defensin 4A | HBD-2 | DEFB4A | 0.122 |
| Plasma | Neurofilament light polypeptide | NFL | NEFL | 0.112 |
| Plasma | D-amino-acid oxidase | OXDA | DAO | 0.100 |
| Saliva | Follicular dendritic cell secreted peptide | FDSCP | FDCSP | 0.089 |
| Plasma | Disintegrin and metalloproteinase domain-containing protein 11 | ADA11 | ADAM11 | 0.071 |
| Plasma | Semaphorin-6A | Semaphorin-6A | SEMA6A | 0.069 |
| Plasma | Endothelial monocyte-activating polypeptide 2 | EMAP-2 | AIMP1 | 0.066 |
| Plasma | Integral membrane protein DGCR2/IDD | IDD | DGCR2 | 0.063 |
| Plasma | Centromere/kinetochore protein zw10 homolog | ZW10 | ZW10 | 0.060 |
| Plasma | Alpha-N-acetylneuraminide alpha-2,8-sialyltransferase | SIA8A | ST8SIA1 | 0.057 |
| Saliva | Glucosylceramidase | GLCM | GBA | 0.054 |
| Plasma | Neuronal regeneration-related protein | NREP | NREP | 0.053 |
| Saliva | COP9 signalosome complex subunit 7b | CSN7B | COPS7B | 0.049 |
| Plasma | Myosin regulatory light chain 2, atrial isoform | MLRA | MYL7 | 0.047 |
| Saliva | Interleukin-36 alpha | IL-1F6 | IL36A | 0.043 |
| Plasma | GDNF family receptor alpha-1 | GFRa-1 | GFRA1 | 0.037 |
| Saliva | B-cell antigen receptor complex-associated protein alpha chain | CD79A | CD79A | 0.035 |
| Plasma | C-C motif chemokine 3 | MIP-1a | CCL3 | 0.033 |
| Plasma | N-acetyl-D-glucosamine kinase | NAGK | NAGK | 0.018 |
| Plasma | Syntaxin-binding protein 6 | STXB6 | STXBP6 | 0.008 |
| Plasma | Nicotinamide/nicotinic acid mononucleotide adenylyltransferase 1 | NMNA1 | NMNAT1 | 0.001 |
| Saliva | Serine protease inhibitor Kazal-type 1 | TATI | SPINK1 | 0.000 |
| Plasma | Obestatin | Obestatin | GHRL | -0.008 |
| Plasma | Apolipoprotein M | ApoM | APOM | -0.014 |
| Plasma | Uncharacterized protein C10orf35 | CJ035 | FAM241B | -0.015 |
| Plasma | Glycodelin | Glycodelin | PAEP | -0.016 |
| Plasma | Nuclear receptor coactivator 2 | NCOA2 | NCOA2 | -0.025 |
| Plasma | Insulin-like growth factor-binding protein 5 | IGFBP-5 | IGFBP5 | -0.048 |
| Plasma | Leucine-rich repeat and fibronectin type-III domain-containing protein 3 | LRFN3 | LRFN3 | -0.053 |
| Plasma | Mth938 domain-containing protein | AAMDC | AAMDC | -0.077 |
| Plasma | Appetite-regulating hormone | ghrelin | GHRL | -0.116 |
| Plasma | Legumain | LGMN | LGMN | -0.198 |
| Plasma | Adiponectin | Adiponectin | ADIPOQ | -0.231 |
| Plasma | Apolipoprotein F | Apo F | APOF | -0.250 |
| Plasma | Secreted and transmembrane protein 1 | SECTM1 | SECTM1 | -0.253 |
| Plasma | Kit ligand | SCF | KITLG | -0.302 |

**Supplement Table 9**. Multi-fluid multi-omics profile for insulin resistance

|  | Protein | | | Metabolite | | | Weight in signature |
| --- | --- | --- | --- | --- | --- | --- | --- |
| Fluid Type | Protein Name | Reported Name or Label | EntrezGeneSymbol | Metabolite Name | Super Pathway | Sub-pathway |  |
| Plasma | Beta-glucuronidase | BGLR | GUSB |  |  |  | 0.326 |
| Plasma |  |  |  | X - 11470 |  |  | 0.288 |
| Plasma | Cyclin-dependent kise 2-interacting protein | CINP | CINP |  |  |  | 0.266 |
| Plasma |  |  |  | X - 12844 |  |  | 0.216 |
| Plasma | Beta-defensin 4A | HBD-2 | DEFB4A |  |  |  | 0.184 |
| Plasma | Neurofilament light polypeptide | NFL | NEFL |  |  |  | 0.162 |
| Plasma | Butyrophilin-like protein 8 | BTNL8 | BTNL8 |  |  |  | 0.141 |
| Plasma | Tissue-type plasminogen activator | tPA | PLAT |  |  |  | 0.140 |
| Plasma | Integral membrane protein DGCR2/IDD | IDD | DGCR2 |  |  |  | 0.132 |
| Plasma | Disintegrin and metalloproteise domain-containing protein 11 | ADA11 | ADAM11 |  |  |  | 0.118 |
| Plasma |  |  |  | X - 11444 |  |  | 0.106 |
| Saliva | Stromelysin-2 | MMP-10 | MMP10 |  |  |  | 0.082 |
| Plasma |  |  |  | cortolone glucuronide (1) | Lipid | Corticosteroids | 0.081 |
| Plasma | D-amino-acid oxidase | OXDA | DAO |  |  |  | 0.080 |
| Plasma | Tubulin-specific chaperone A | TBCA | TBCA |  |  |  | 0.070 |
| Saliva | Follicular dendritic cell secreted peptide | FDSCP | FDCSP |  |  |  | 0.070 |
| Plasma | C-C motif chemokine 3 | MIP-1a | CCL3 |  |  |  | 0.061 |
| Plasma | Pigment epithelium-derived factor | PEDF | SERPINF1 |  |  |  | 0.059 |
| Plasma | Myosin regulatory light chain 2, atrial isoform | MLRA | MYL7 |  |  |  | 0.054 |
| Plasma | Liver-expressed antimicrobial peptide 2 | LEAP2 | LEAP2 |  |  |  | 0.053 |
| Plasma |  |  |  | 2'-deoxyuridine | Nucleotide | Pyrimidine Metabolism, Uracil containing | 0.049 |
| Plasma | Amyloid beta A4 precursor protein-binding family B member 3:Phosphotyrosine Interaction Domain 2, Isoform IV | APBB3:PID 2 | APBB3 |  |  |  | 0.044 |
| Saliva | Serine protease inhibitor Kazal-type 1 | TATI | SPINK1 |  |  |  | 0.040 |
| Plasma | Centromere/kinetochore protein zw10 homolog | ZW10 | ZW10 |  |  |  | 0.038 |
| Plasma | Pre-miR 5'-monophosphate methyltransferase | BN3D2 | BCDIN3D |  |  |  | 0.036 |
| Plasma |  |  |  | (2-butoxyethoxy)acetic acid | Xenobiotics | Chemical | 0.034 |
| Saliva | Prolactin-inducible protein | PIP | PIP |  |  |  | 0.026 |
| Plasma |  |  |  | X - 24953 |  |  | 0.023 |
| Plasma | Gap junction alpha-8 protein | CXA8 | GJA8 |  |  |  | 0.021 |
| Saliva | StAR-related lipid transfer protein 5 | STAR5 | STARD5 |  |  |  | 0.019 |
| Plasma |  |  |  | X - 24432 |  |  | 0.018 |
| Saliva | Absent in melanoma 2 | AIM2 | AIM2 |  |  |  | 0.018 |
| Plasma | GDNF family receptor alpha-1 | GFRa-1 | GFRA1 |  |  |  | 0.009 |
| Saliva | B-cell antigen receptor complex-associated protein alpha chain | CD79A | CD79A |  |  |  | 0.009 |
| Plasma | BTB/POZ domain-containing protein KCTD2 | KCTD2 | KCTD2 |  |  |  | 0.008 |
| Plasma | Galectin-2 | Galectin-2 | LGALS2 |  |  |  | 0.006 |
| Plasma | N-acetyl-D-glucosamine kise | GK | GK |  |  |  | 0.002 |
| Saliva | Protein LEG1 homolog | CF058 | LEG1 |  |  |  | 0.001 |
| Plasma | Fc_MOUSE | Fc_MOUSE |  |  |  |  | 0.000 |
| Plasma | Leucine-rich repeat and fibronectin type-III domain-containing protein 3 | LRFN3 | LRFN3 |  |  |  | -0.007 |
| Plasma | GRB2-related adapter protein 2 | GRB2-related adapter protein 2 | GRAP2 |  |  |  | -0.007 |
| Plasma | Type 2 lactosamine alpha-2,3-sialyltransferase | SIA10 | ST3GAL6 |  |  |  | -0.010 |
| Plasma | Glycodelin | Glycodelin | PAEP |  |  |  | -0.016 |
| Plasma | Mth938 domain-containing protein | AAMDC | AAMDC |  |  |  | -0.021 |
| Plasma | Matrix-remodeling-associated protein 8:Extracellular domain | MXRA8:ECD | MXRA8 |  |  |  | -0.030 |
| Plasma | Insulin-like growth factor-binding protein 5 | IGFBP-5 | IGFBP5 |  |  |  | -0.036 |
| Plasma |  |  |  | X - 13729 |  |  | -0.041 |
| Plasma | Glycosyltransferase 8 domain-containing protein 1 | GL8D1 | GLT8D1 |  |  |  | -0.050 |
| Plasma | Human Chorionic Godotropin | HCG | CGA\|CGB3\|CGB7 | |  |  | -0.053 |
| Plasma | Potassium voltage-gated channel subfamily E regulatory beta subunit 5:Cytoplasmic domain | KCE1L:CD | KCNE5 |  |  |  | -0.056 |
| Plasma |  |  |  | glutarylcarnitine (C5-DC) | Amino Acid | Lysine Metabolism | -0.059 |
| Plasma |  |  |  | 1-(1-enyl-stearoyl)-2-oleoyl-GPE (P-18:0/18:1) | Lipid | Plasmalogen | -0.060 |
| Plasma | Adiponectin | Adiponectin | ADIPOQ |  |  |  | -0.102 |
| Plasma | Appetite-regulating hormone | ghrelin | GHRL |  |  |  | -0.139 |
| Plasma | Secreted and transmembrane protein 1 | SECTM1 | SECTM1 |  |  |  | -0.207 |
| Plasma | Legumain | LGMN | LGMN |  |  |  | -0.213 |
| Plasma | Apolipoprotein F | Apo F | APOF |  |  |  | -0.283 |
| Plasma | Kit ligand | SCF | KITLG |  |  |  | -0.297 |
| All metabolites beginning with “X –“ are unknown metabolites. The number assigned to each unknown was provided by Metabolon, Inc. | | | | | | | |

**Supplement Table 10**. Predication ability across signatures

| Signature | AUC | 95%CI_Lower bound | 95%CI_Upper bound | Permutation P value |
| --- | --- | --- | --- | --- |
| Plasma metabolomic signature | 0.610 | 0.442 | 0.820 | 0.33 |
| Plasma proteomic signature | 0.823 | 0.675 | 0.940 | 0.002 |
| Plasma multiomic signature | 0.830 | 0.667 | 0.958 | 0.002 |
| Saliva metabolomic signature | 0.650 | 0.452 | 0.823 | 0.15 |
| Saliva proteomic signature | 0.672 | 0.507 | 0.850 | 0.06 |
| Saliva multiomic signature | 0.510 | 0.465 | 0.710 | 0.81 |
| Multifluid metabolomic signature | 0.585 | 0.442 | 0.792 | 0.43 |
| Multifluid proteomic signature | 0.802 | 0.655 | 0.927 | 0.003 |
| Multifluid multiomic signature | 0.812 | 0.645 | 0.945 | 0.003 |

AUC, area under the curve
